# Comparative efficacy of different virtual reality-based interventions for static balance, dynamic balance, and fear of falling in older adults: a systematic review and network meta-analysis

**DOI:** 10.3389/fpubh.2026.1973306

**Published:** 2026-10-20

**Authors:** Meng Tian, Qiang Chen, Tianwei Lou

**Affiliations:** 1 Shandong Institute of Sport Science, Jinan, China; 2 Shandong University of Traditional Chinese Medicine, Jinan, China; 3 Shandong Public Health Clinical Center, Jinan, China; 4 Affiliated Hospital of Shandong University of Chinese Medicine, Jinan, China

**Keywords:** balance, fear of falling, network meta-analysis, older adults, virtual reality

## Abstract

**Background and objectives:**

Virtual reality (VR) is increasingly used to improve balance and reduce fear of falling in older adults, but comparative effectiveness of different VR types remains unclear. This network meta-analysis (NMA) aimed to compare and rank the efficacy of various VR interventions against usual care for static balance, dynamic balance, and fear of falling.

**Methods:**

Two investigators independently searched Embase, PubMed, PsycINFO, Cochrane Library, CNKI, and VIP for RCTs published from inception to May 30, 2026. Eligible studies included older adults receiving VR versus usual care or active control, with outcomes measured by validated tools. Risk of bias was assessed using RoB 2. A random-effects consistency model was applied, using MDs or SMDs with 95% CIs.

**Results:**

Nineteen RCTs (1,815 participants) were included. For static balance, Kinect (MD = 6.83, 95% CI: 1.78–11.89), IVR (6.80, 0.28–13.30), and Wii (3.89, 0.34–7.44) significantly outperformed UC; SUCRA rankings placed Kinect first (82.2%), followed by IVR (78.1%) and BTSNVR (56.8%). For dynamic balance, BTSNVR (SMD = −3.66, −5.22 to −1.20), Wii (−2.79, −3.93 to −1.66), Kinect (−1.87, −2.53 to −1.22), and AC (−1.66, −2.35 to −0.97) were superior to UC; SUCRA ranked BTSNVR best (87.5%), Wii (82.0%), and Kinect (57.1%). For fear of falling, only Kinect showed significant reduction (SMD = −3.52, −5.48 to −1.56), ranking highest (78.7%), with IVR (66.3%) and BTSNVR (60.9%) less effective. However, several comparisons were based on few studies, so findings are preliminary.

**Conclusion:**

Specific VR interventions offer distinct advantages: Kinect may benefit static balance and fear reduction, while BTSNVR improves dynamic balance. Rankings should be interpreted cautiously. Larger head-to-head RCTs with standardized protocols and longer follow-up are needed to validate these findings and establish clinical recommendations.

**Systematic review registration:**

https://www.crd.york.ac.uk/PROSPERO/view/CRD420261464816, CRD420261464816.

## 1 Introduction

Falls are one of the primary public health issues threatening the health and quality of life of older adults worldwide. According to the Global Burden of Disease Study, approximately 696,000 deaths were caused by falls globally in 2017 (1), while in the United States, about 27.6% of adults aged 65 years and older reported experiencing falls in the previous year (2). Falls can lead to direct physical injuries such as fractures and traumatic brain injuries; however, they can also trigger a fear of falling again, and such fear often causes older adults to voluntarily restrict their daily activities and avoid social interactions. These behavioral changes ultimately accelerate any decline in muscle strength and balance function, which can further increase the risk of future falls. This forms a vicious cycle of physical injury, psychological disorder, and functional decline (3, 4). Balance dysfunction is the a central risk factor for falls, and exploring safe and effective rehabilitation approaches for balance function has thus become an important research topic in the fields of both geriatrics and rehabilitation medicine (5, 6).

The current large body of evidence suggests that regular exercise training is an effective nonpharmacological intervention strategy for improving balance function and reducing fall risk in older adults (7, 8). However, traditional balance function training typically includes strength training, proprioceptive training, coordination training, and gait training, as well as needing to be conducted under the guidance of professional rehabilitation institutions or therapists. This approach places high levels of demand on professional staff, venues, and equipment, as well as being dependent of transportation accessibility. Such approaches thus face multiple constraints in terms of clinical promotion and community application. There are significant differences in the distribution of professionals such as physiotherapists across different regions, and accessibility of rehabilitation services is much lower in rural areas than in urban areas (9, 10). A cross-sectional geographic information system (GIS) study of Texas in the United States showed that the ratio of general population to rehabilitation therapists ranged from 4:1 to 11,147:1 across census tracts, indicating marked disparities (9). Overall, the number of physical therapists in rural areas is approximately 40% lower than in urban areas (11). Meanwhile, older adults also face multiple barriers to participating in and adhering to regular exercise, including fear of injury, pain, lack of motivation, insufficient social support, environmental inconvenience, and financial burdens (12, 13). One 12-month prospective study showed that the proportion of older adults maintaining high adherence to such programs was only 51.3% at 6 months, which decreased to 47.8% at 12 months (14). The interplay of these factors has led to a generally low level of participation, enthusiasm, and training compliance in traditional balance training programs, seriously limiting their widespread adoption and reducing the maintenance of long-term benefits.

A wide variety of virtual reality (VR) systems are now being used in older adults (15, 16). Based on the provided level of immersion, these systems can be categorized into nonimmersive VR systems and immersive VR systems. The former typically present virtual scenarios on televisions or projection screens, whereas the latter use head-mounted displays to provide a more fully immersive visual experience (17, 18). Within the nonimmersive VR category, several commercial motion-sensing game platforms are widely used for balance training in older adults. Based on differences in interactive hardware and feedback mechanisms, these platforms can be further divided into balance-board-based systems and motion-capture-based systems. A meta-analysis by Cieślik et al. systematically compared these two types of systems to determine that motion-capture-based systems produced significantly superior results as compared to usual treatment and no-treatment control groups with respect to improving balance function in older adults (19). A meta-analysis by Liu et al. that included 10 studies confirmed that training based on the Wii balance board platform significantly improved Berg Balance Scale (BBS) scores and Timed Up and Go (TUG) test results in older adults (20). A further network meta-analysis by Cieślik et al. that included 52 studies categorized technology-assisted interventions, identifying exergames, serious games, wearable devices, and telerehabilitation, amongst others. This study also found that motion-capture-based exergames produced significantly superior results as compared to no-treatment control groups regarding improving functional mobility in older adults (21). However, the intervention classification in the network meta-analysis was relatively broad and did not further distinguish efficacy differences across specific device types such as the Kinect and Wii.

Although existing evidence generally supports the value of VR training in terms of improving balance function in older adults, several important limitations must be urgently addressed. First, most previous meta-analyses have focused on pairwise comparisons between VR training and conventional training or no intervention, leading to a lack of head-to-head direct comparisons between different VR systems. Second, different VR systems differ fundamentally across various areas such as immersion degree, interaction modality, feedback type, and training task design. These differences in technical parameters may lead to significant divergences in therapeutic mechanisms. Third, although preliminary network meta-analyses have explored the relative effectiveness of VR-assisted rehabilitation, no study to date has systematically compared the relative efficacy rankings of mainstream VR intervention modalities such as Kinect, IVR, Wii, BTS Nirvana, and BioRescue in terms of improving BBS, TUG, and fear of falling in older adults. This evidence gap leaves clinicians without evidence-based guidance for the multiple VR device options.

Based on this, this study aimed to use a network meta-analysis to systematically compare the effectiveness of five VR intervention modalities, namely Kinect, IVR, Wii, BTSNVR, and BioRescue, with respect to improving balance function and the ability to stand and walk, and reducing fear of falling in older adults. The surface under the cumulative ranking curve (SUCRA) was thus used to rank the efficacy of each intervention. The findings of this study are intended to provide evidence-based medical evidence for the optimal selection of VR intervention programs for the older adult population, and to offer a reference for the updating and refinement of relevant clinical practice guidelines.

## 2 Methods

This systematic review and network meta-analysis were registered with the International Prospective Register of Systematic Reviews (CRD420261464816). The review was conducted in accordance with the Preferred Reporting Items for Systematic Reviews and Meta-Analyses for Network Meta-Analyses (PRISMA-NMA) statement (22).

### 2.1 Search strategy

Two investigators (M. T. and Q. C.) searched six databases, Embase, PubMed, PsycINFO, the Cochrane Library, the China National Knowledge Infrastructure (CNKI), and the Chinese Scientific Journal Database (VIP), to identify relevant randomized controlled trials (RCTs) published from database inception in each case to May 30, 2026. The detailed search strategy used for the PubMed case is presented in Supplementary Table S1.

### 2.2 Inclusion and exclusion criteria

The inclusion criteria were formulated based on the PICOS (Participants, Interventions, Comparisons, Outcomes, Study Design) framework in order to screen for eligible studies:

Participants: Older adults (age ≥ 60 years) were included.
Interventions: Participants in the intervention group received VR training (such training administered on top of control interventions was also eligible).
Comparisons: Participants in the control group received alternative VR training, active control (conventional exercise training), or usual care control (routine care without structured exercise training or no intervention).
Outcomes: Outcomes included measures of static balance, dynamic balance, and fear of falling using standardized and validated assessment tools.
Study design: Only randomized controlled trials (RCTs) published in English or Chinese were included.

The exclusion criteria were as follows:

1. Studies exclusively recruiting older patients with neurological disorders, cancer, or other relevant illnesses;
2. Study protocols, conference abstracts, commentaries, duplicate publications, retracted articles, and studies without full-text availability;
3. Studies with no accessible or convertible extractable data.

### 2.3 Data extraction and article selection

Two investigators (M. T. and Q. C.) independently extracted data using a prespecified structured data extraction form. The extracted information included study characteristics (authors, publication year, country, group allocation, sample size, etc.), demographic characteristics (e.g., participant age), details of VR training (such as type, duration, frequency and session cycle), control group interventions, and outcome measures (static balance, dynamic balance, fear of falling). When data from eligible studies could not be pooled, the corresponding and first authors of the relevant publications were contacted to obtain the required data.

### 2.4 Assessment of the transitivity assumption

We assessed the plausibility of the transitivity assumption by descriptively comparing the distributions of potential effect modifiers across the included studies. The factors examined included residential or care setting, sex, age, cognitive status, baseline balance function, intervention delivery mode, training duration, training frequency, training period, and control intervention content. For intervention delivery mode, we considered whether the intervention was standalone or added to a common treatment. Together with the study characteristics, we examined the overlap and differences in these characteristics across intervention comparisons, focusing on systematic imbalances that could affect relative intervention effects.

### 2.5 Risk of bias assessment and quality of evidence

Two investigators (M. T. and Q. C.) independently assessed the methodological quality of included studies using the Cochrane risk-of-bias tool for randomized trials (Version 2, RoB 2). This tool evaluates six key domains of studies, namely the randomization process, adherence to the intervention, missing outcome data, outcome measurement, selective reporting, and overall risk of bias. Each area was rated as being at a “low,” “unclear,” or “high” risk of bias. Disagreements were resolved through adjudication by a third reviewer (T. L.). We used the Confidence in Network Meta-Analysis framework (CINeMA) to assess the confidence in the evidence from the network meta-analysis with respect to within-study bias, reporting bias, indirectness, imprecision, heterogeneity, and incoherence (23).

### 2.6 Statistical analysis

Network meta-analysis was conducted using Stata 16.0 software. Due to heterogeneity among the various VR training interventions included, frequentist network meta-analysis was implemented in Stata 16.0 with a random-effects model. For this network meta-analysis, the similarity assumption was judged by assessing clinical and epidemiological effect modifiers (24) such as mean age in included RCTs. A consistency network meta-analysis model was then fitted using the restricted maximum likelihood estimation procedure (25, 26). The global inconsistency was evaluated with the design-by-treatment interaction test, and the node-splitting method was used to further test local inconsistency (27, 28). A *p* value ≥ 0.05 was deemed to suggest the absence of significant global inconsistency; in such cases, the consistency model was employed. Otherwise, the inconsistency model was adopted. If closed loops were formed in the intervention network plots, loop-specific methods were used to test loop inconsistency, and significant inconsistency within a loop was identified when the *p* value < 0.05.

Standardized mean differences (SMDs) with 95% confidence intervals (CIs) were adopted to compare relative effectiveness where the included studies measured the effects of VR training using different assessment tools. League table analyses were conducted to present comparisons across multiple VR training regimens from the network meta-analysis. Probabilistic ranking based on the surface under the cumulative ranking curve (SUCRA) method was constructed to identify the optimal VR training type. Comparative-adjusted funnel plots were also used to detect publication bias and small-study effects when at least 10 studies were included in a category (29).

For direct comparisons with at least two studies, we performed pairwise meta-analyses using a random-effects model. We calculated pooled effect sizes and their 95% confidence intervals. We assessed between-study heterogeneity using the I^2^ statistic. We further evaluated the robustness of the results through sensitivity analyses. In addition, we conducted a qualitative analysis. We incorporated the characteristics of the included studies and discussed the potential influence of these factors on intervention effects.

## 3 Results

### 3.1 Study selection and characteristics

A total of 1,638 records were retrieved from the six databases and Supplementary material. After removal of 673 duplicate records, the remaining 965 citations underwent initial screening, leaving 61 articles for full-text eligibility assessment. Ultimately, 19 RCTs were included in the study (30–43) (Table 1). The study screening process and reasons for exclusion are illustrated in the PRISMA flow diagram in Figure 1.

**Table 1:** Summary of characteristics of the included studies.

Study	Country	Age (Mean ± SD)	Sample size	Intervention (duration, frequency and cycle)	Control	Outcomes
Babadi and Daneshmandi (30)	Iran	Kinect: 66.5 ± 3.8 AC: 67.5 ± 3.11 UC: 66.75 ± 3.27	Kinect: 12 AC: 12 UC: 12	Kinect-based VR training, 60 min/session, 3 times/week for 9 weeks	AC: conventional balance training UC: no specialized balance training	TUG
Campo-Prieto et al. (31)	Spain	IVR: 85.08 ± 8.48 UC: 84.82 ± 8.10	IVR: 13 UC: 11	Immersive VR training, 6 min/session, 3 times/week for 10 weeks	UC: no specialized balance training	TUG
Delbroek et al. (32)	Belgium	BioRescue: 86.9 ± 5.6 UC: 87.5 ± 6.6	BioRescue: 8 UC: 9	BioRescue VR training, 18–30 min/session, 2 times/week for 6 weeks	UC: no specialized balance training	TUG
Fakhro et al. (33)	Lebanon	Total: 72.2 ± 5.2	Wii: 30; UC: 30	Wii-based VR training, 40 min/session, 3 times/week for 8 weeks	UC: no specialized balance training	TUG
Gomes et al. (34)	Brazil	Wii: 83 ± 5.87 UC: 85 ± 6.19	Wii: 13 UC: 13	Wii-based VR training, 50 min/session, 2 sessions /week for 7 weeks	UC: no specialized balance training	FES-I
Jung et al. (35)	South Korea	Wii: 74.3 ± 2.1 AC: 74.3 ± 3.5 UC: 73.6 ± 2.4	Wii: 8 AC: 8 UC: 8	Wii-based VR training, 30 min/session, 2 sessions /week for 8 weeks	AC: conventional balance training UC: no specialized balance training	BBS, TUG
Kwok et al. (36)	Singapore	Wii: 70.5 ± 6.7 AC: 69.8 ± 7.5	Wii: 40 AC: 40	Wii-based VR training, 60 min/session, 1 session/week for 12 weeks	AC: conventional balance training	TUG
Lee et al. (37)	South Korea	Kinect:68.77 ± 4.62 AC: 67.71 ± 4.31	Kinect: 26 AC: 28	Kinect-based VR training, 60 min/session, 3 times/week, for 8 weeks	AC: conventional balance training	8-Foot Up-and- Go Test
Liao et al. (38)	China	Kinect:79.6 ± 8.5 AC: 84.1 ± 5.5	Kinect: 27 AC: 25	Kinect-based VR training, 60 min/session, 3 sessions/week for 12 weeks	AC: conventional balance training	TUG, FES
Merriman et al. (39)	Ireland	Total: 74 (range ≥60)	Wii:38 UC: 38	Wii-based VR training, 30 min/session, 2 sessions/week for 5 weeks	UC: no specialized balance training	BBS
Molhemi et al. (40)	Iran	Kinect: 63.8 ± 8.4 AC: 61.6 ± 8.4	Kinect: 19 AC: 20	Kinect-based VR training, 30 min/session, 3 sessions/week for 6 weeks	AC: conventional balance training	BBS, FES, TUG
Montero-Alía et al. (41)	Spain	Median age: 75.3	Wii: 508 UC: 469	Wii-based VR training, 30 min/session, 2 sessions/week for 12 weeks	UC: no specialized balance training	FES
Padala et al. (42)	USA	Wii: 79.3 ± 9.8 AC: 81.6 ± 5.2	Wii: 11 AC: 11	Wii-based VR training, 30 min/session, 5 sessions/week for 8 weeks	AC: conventional balance training	BBS, TUG
Parmak et al. (48)	Turkey	IVR: 71.14 ± 4.83 AC: 75.36 ± 9.16	IVR:22 AC: 22	Immersive VR training, 35 min/session, 3 sessions/week for 8 weeks	AC: conventional balance training	FES
Stanmore et al. (43)	UK	Kinect: 77.9 ± 8.9 AC: 77.8 ± 10.2	Kinect: 56 AC: 50	Kinect-based VR training, 3 sessions/week for 12 weeks	AC: conventional balance training	BBS, FES, TUG
Tsang and Fu (44)	China	Wii: 82.3 ± 3.8 AC: 82.0 ± 4.3	Wii: 39 AC: 40	Wii-based VR training, 60 min/session, 3 sessions/week for 6 weeks	AC: conventional balance training	BBS, TUG
Yeşilyaprak et al. (45)	Turkey	BTSNVR: 70.1 ± 4.0 AC: 73.1 ± 4.5	BTSNVR: 7 AC: 11	BTS NIRVANA VR training, 40–50 min/session, 3 sessions/week for 6 weeks	AC: conventional balance training	BBS, FES, TUG
Yoo et al. (46)	South Korea	IVR: 72.90 ± 3.41 AC: 75.64 ± 5.57	IVR: 10 AC: 11	Immersive VR training, 60 min/session, 3 sessions/week for 12 weeks	AC: conventional balance training	BBS
Zahedian-Nasab et al. (47)	Iran	Kinect: 69.67 ± 7.72 AC: 72.00 ± 7.81	Kinect: 30 AC: 30	Kinect-based VR training, 30–60 min/session, 2 sessions/week for 6 weeks	AC: conventional balance training	BBS, FES, TUG

AC, Active Control; UC, Usual Control; TUG, Timed Up and Go; BBS, Berg Balance Scale; FES, Falls Efficacy Scale; FES-I, Falls Efficacy Scale-International; IVR, Immersive VR.

**Figure 1:**
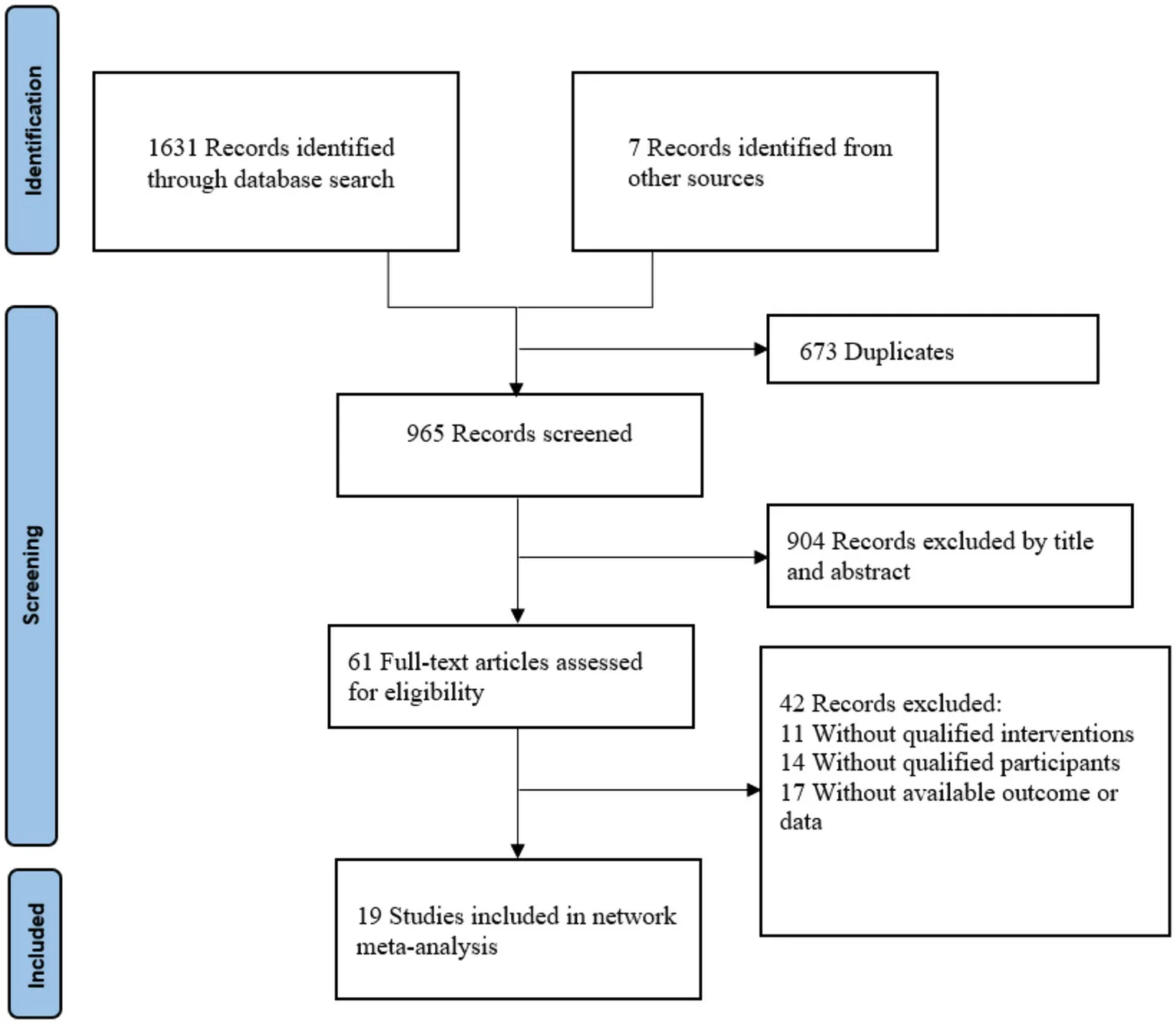
PRISMA flowchart.

Across the 19 included studies (30–43), a total of 1,815 participants received five types of VR training and two control interventions. The VR training modalities were Nintendo Wii Fit-based VR Training (Wii), Microsoft Kinect-based VR Training (Kinect), Immersive VR Training (IVR), BTS NIRVANA VR Interactive System (BTSNVR), and BioRescue VR Platform (BioRescue). Detailed information regarding VR training protocols in the included studies is shown in Supplementary Table S2. Control groups were classified into active control (AC; participants received conventional balance training) and usual care control (UC; participants received no intervention or only routine care without structured exercise) types.

Among the 19 included studies, nine (25, 39, 40, 42, 43–47) reported the effects of four types of VR training on static balance in older adults; fourteen studies (30–33, 35–38, 40, 42, 43–46) described the impacts of five types of VR training on dynamic balance in older adults; and eight (34, 38, 40, 41, 43, 45–48) assessed the effects of four types of VR training on the severity of fear of falling among older adults.

### 3.2 Risk of bias among included trials

Among the included studies, 5.3% (1/19) were judged to have low risk of bias (33), whereas 94.7% (18/19) were rated as causing some concerns (30–32, 34–38). Ten studies (52.6%) (31, 32, 34, 36–42) were assigned some concerns in the domain of deviations from intended interventions, which is potentially attributable to the difficulty of blinding participants during VR training. Furthermore, ten studies (52.6%) (30, 31, 35, 41, 42, 43, 44, 46–48) received some concerns for the outcome measurement domain, as most lacked information regarding assessor blinding (Supplementary Figure S1).

### 3.3 Transitivity assessment

We assessed the plausibility of transitivity by descriptively comparing the distributions of potential effect modifiers across included studies (Supplementary Table S3). Wii, Kinect, and IVR studies involved community and institutional populations, whereas BioRescue and BTS NIRVANA focused on institutional residents. For cognitive status, most studies included participants who could understand the intervention protocol, while a few did not report this information. Baseline balance function also differed across studies. Interventions included standalone training or training added to common care. Training duration, frequency, and period varied. Overall, studies overlapped in several population and intervention characteristics. This overlap supported the clinical comparability of relevant indirect comparisons and the plausibility of transitivity. However, imbalanced distributions of potential effect modifiers could not be fully ruled out, especially for comparisons supported by only one study or with inadequate reporting of key characteristics. Therefore, the evidence supported the plausibility of transitivity, but indirect effect estimates and intervention rankings should be interpreted cautiously given these uncertainties.

### 3.4 Results of the network meta-analysis

#### 3.4.1 Network plots

The network plots illustrate the comparative connections among VR training interventions for balance function in older adults across included trials (Figure 2). Each node represents one intervention, and the size of each node corresponds to the sample size. Lines connecting nodes indicate direct comparisons between two interventions, with line thickness being proportional to the number of studies evaluating the paired interventions. The network plot for static balance contained one closed loop, which originated from a single three-arm trial, and the network plot for dynamic balance formed two closed loops.

**Figure 2:**
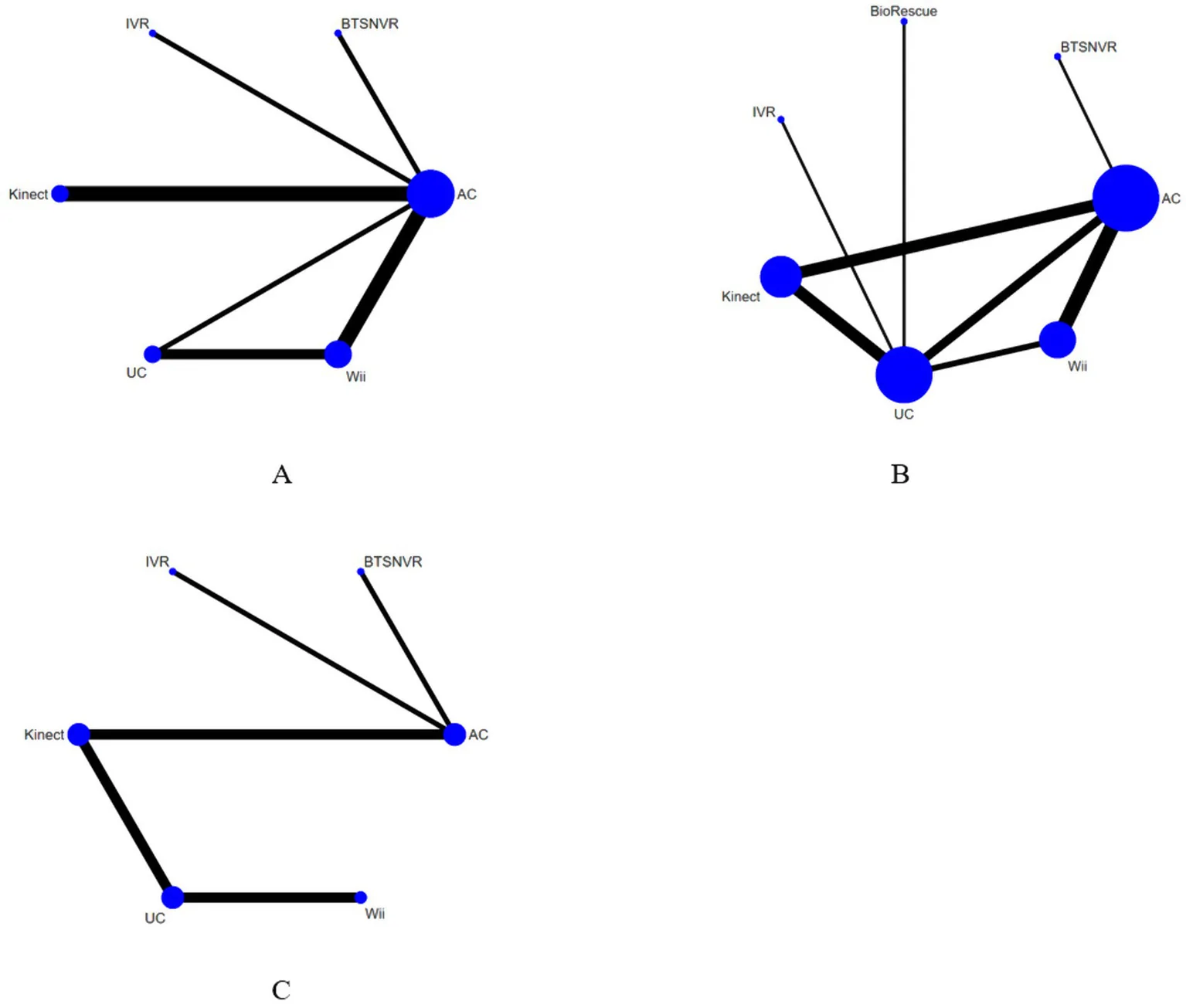
Network plots for static balance **(A)**, dynamic balance **(B)**, and fear of falling **(C)**.

#### 3.4.2 Inconsistency testing and publication bias

In the network meta-analysis of dynamic balance, the design-by-treatment interaction test revealed no statistically significant global inconsistency (*p* = 0.459). Node-splitting analyses showed all *p* values > 0.05, indicating the absence of statistically significant inconsistency between direct and indirect comparisons. The consistency model was therefore adopted. The closed loop observed in the network meta-analysis for static balance stemmed from a single three-arm trial, and no closed loops were formed in the network for fear of falling. Accordingly, consistency models were applied for both outcomes. Loop-specific tests were performed for the network meta-analysis of dynamic balance. The results showed that all 95% confidence intervals (CIs) encompassed zero (*p* > 0.05), suggesting no significant loop inconsistency for dynamic balance (Supplementary Figure S2). Additionally, the comparative-adjusted funnel plot for dynamic balance was roughly symmetric around the central line, implying no substantial publication bias (Figure 3).

**Figure 3:**
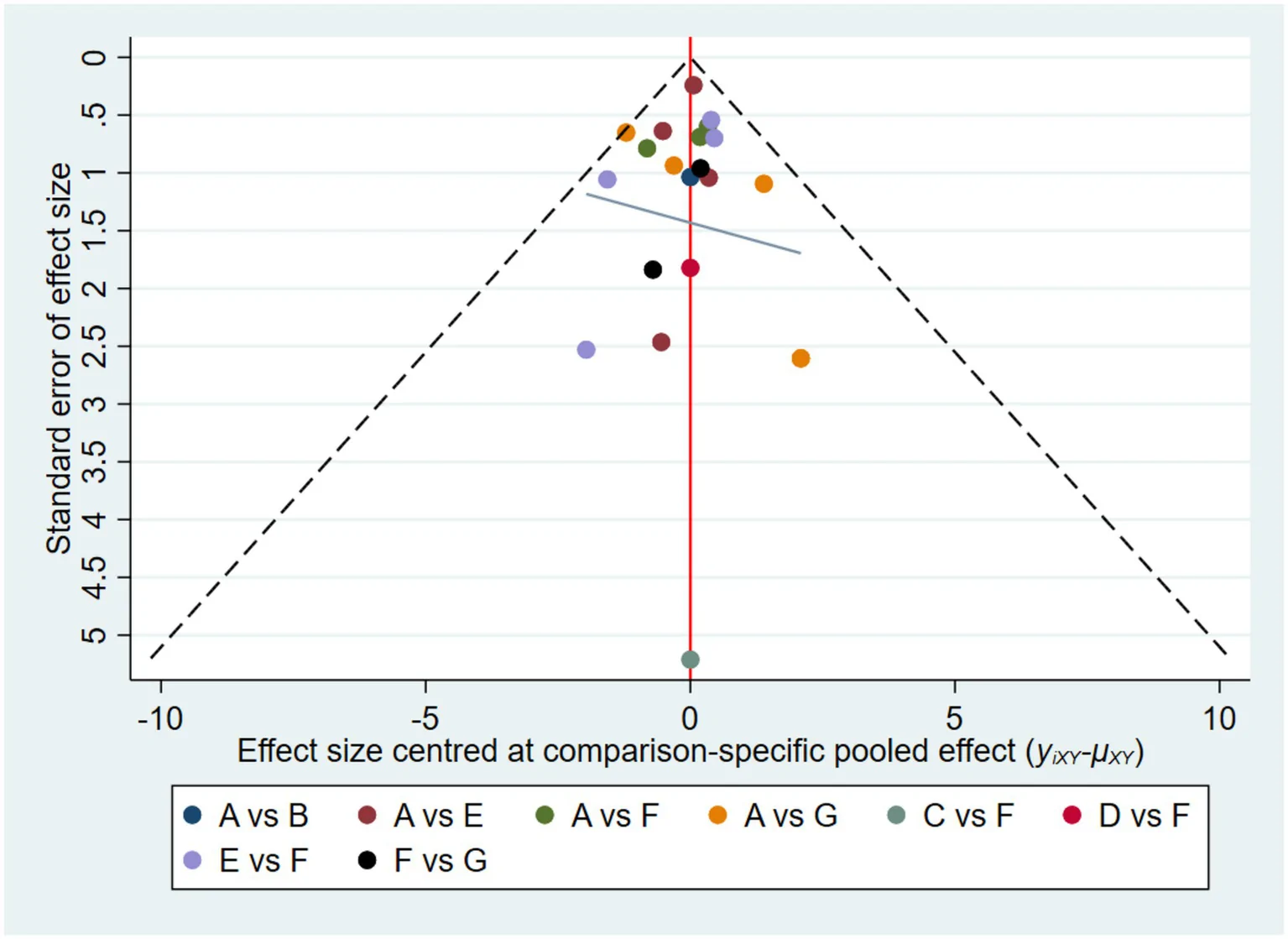
Funnel plots for dynamic balance.

#### 3.4.3 Effectiveness and ranking of static balance

Under the consistency model, the findings of network meta-analysis revealed that, compared with the UC control group, Kinect (MD = 6.83, 95% CI: 1.78–11.89), IVR (MD = 6.80, 95% CI: 0.28–13.30), and Wii (MD = 3.89, 95% CI: 0.34–7.44) significantly improved static balance among older adults (Supplementary Table S4).

Efficacy rankings generated using the SUCRA approach placed Kinect (82.2%), IVR (78.1%), and BTSNVR (56.8%) as the top three interventions. The remaining rankings were Wii (42.8%), AC (37.5%), and UC (2.6%).

#### 3.4.4 Effectiveness and ranking of dynamic balance

Under the consistency model, the network meta-analysis demonstrated that use of BTSNVR (SMD = −3.66, 95% CI: −5.22−−1.20), Wii (SMD = −2.79, 95% CI: −3.93−−1.66), Kinect (SMD = −1.87, 95% CI: −2.53−−1.22), and AC (SMD = −1.66, 95% CI: −2.35−−0.97) showed significant improvements in dynamic balance among older adults as compared with the UC control group (Supplementary Table S5).

Efficacy rankings derived from the SUCRA approach showed BTSNVR (87.5%), Wii (82.0%), and Kinect (57.1%) to be the top three interventions. The remaining rankings were AC (46.5%), BioRescue (33.5%), IVR (25.9%), and UC (17.1%).

#### 3.4.5 Effectiveness and ranking of fear of falling

Under the consistency model, network meta-analysis results indicated that compared with the UC control group, Kinect (SMD = −3.52, 95% CI: −5.48−−1.56) significantly reduced fear of falling in older adults (Supplementary Table S6).

Efficacy rankings generated by the SUCRA approach identified Kinect (78.7%), IVR (66.3%), and BTSNVR (60.9%) as the top three interventions. The remaining rankings were AC (50.9%), Wii (28.4%), and UC (14.8%).

### 3.5 Assessment of evidence quality

For the static balance outcome, we assessed 15 intervention comparisons, of which 3 were low certainty and 12 were very low certainty. The main reason for downgrading was incoherence. The evidence for Wii versus UC and AC was low certainty. For the dynamic balance outcome, we assessed 21 intervention comparisons, of which 2 were moderate certainty, 3 were low certainty, and 16 were very low certainty. Most very low certainty comparisons were supported by indirect evidence only. The main reason for downgrading was incoherence. The moderate and low certainty evidence involved comparisons of Kinect and Wii with control groups. For the fear of falling outcome, we assessed 15 intervention comparisons, all of which were moderate certainty. The main reason for downgrading was within-study bias. Full assessment results are in Supplementary Tables S7–S9.

### 3.6 Sensitivity analysis and qualitative analysis

After we separately excluded studies involving people with baseline balance impairment and people with a history of falls, heterogeneity measured by the I^2^ statistic decreased for some direct comparisons in the static balance, dynamic balance, and fear of falling outcomes (Supplementary Table S10). This finding suggests that these studies may be one source of the observed heterogeneity. It may also be related to differences in population characteristics. Sensitivity analyses did not change the statistical significance of the meta-analysis results. This finding indicates that the current pooled results were relatively robust to excluding these studies. However, sensitivity analyses cannot directly test effect modification. Therefore, we cannot rule out a potential effect-modifying role of these factors. Given the limited number of available studies, future studies are needed to clarify the direction and magnitude of their effects.

### 3.7 Adverse events

Among the 19 included studies, only 4 reported information on adverse events or safety. Campo-Prieto et al. (29) reported no adverse events, and the simulator sickness questionnaire showed no symptoms. Gomes et al. (32) reported few adverse events during Wii fit plus training and considered the intervention safe for frail older adults. Delbroek et al. (30) reported that sadness, anger, and anxiety were almost never experienced during BioRescue training. The remaining 15 studies did not report any adverse event information, including motion sickness, dizziness, cybersickness, or falls during training.

## 4 Discussion

This is the first network meta-analysis to systematically compare the effects of different types of VR interventions, namely Kinect, IVR, Wii, BTSNVR, and BioRescue, on static balance, dynamic balance, and fear of falling in older adults. The main findings are reported below.

In terms of improving static balance, Kinect, IVR, and Wii were all significantly superior to UC, with Kinect taking top ranking (SUCRA = 82.2%). With respect to improving dynamic balance, BTSNVR, Wii, Kinect, and AC were all significantly superior to UC, with BTSNVR taking top ranking (SUCRA = 87.5%). Finally, when considering improving fear of falling, Kinect was significantly superior to UC, taking top ranking (SUCRA = 78.7%). These results indicate that different types of VR interventions each have their own advantages with regard to improving balance function and indicators related to the fear of falling in older adults; treatment selection should thus be based on specific rehabilitation goals.

This study found that Kinect-based exergames ranked top in terms of improving static balance function and alleviating fear of falling, and ranked among the top three with respect to improving dynamic balance function. Kinect-based exergames can thus improve overall balance function in older adults. Kinect is based on markerless motion capture technology, using an infrared depth sensor to track whole-body movements in real time. This allows older adults to interact naturally with virtual scenes without wearing sensors, as it can still track their body movements (49). Kinect-based exergames activate neural pathways related to postural control by means of both multisensory feedback and task-oriented training. They also promote sensorimotor integration and thus improve balance function, while engaging game design enhances motivation to participate. This enables older adults to repeatedly practice balance tasks in a safe virtual environment and thus to gradually reduce their fear of falling (47). Multiple RCTs have confirmed that Kinect interventions can significantly improve balance function and reduce fear of falling in older adults (49, 50). Its contactless nature and low cognitive load make this system suitable even for those older adults who are very old or frail. Compared with IVR, Kinect has advantages across equipment cost, risk of motion sickness, and social support, offering higher convenience and accessibility. It is thus especially suitable for promotion and application in community or home rehabilitation scenarios (51, 52). Those aged 60 years and above are generally able to complete Kinect operations. Training adherence is high, and safety profiles are good, with only occasional transient muscle soreness and no serious adverse events (47). Related studies have been successfully implemented in community senior centers (53), nursing homes (54), and home environments (55), providing direct evidence for the wider promotion of such facilities.

IVR ranked second in terms of both improving static balance function and alleviating fear of falling, suggesting that it also has significant therapeutic effects in these areas. IVR creates a highly immersive virtual environment through the use of head-mounted displays. Multisensory feedback and task-oriented design provide strong ecological validity and presence and activate multiple brain regions related to spatial navigation, postural control, and anticipatory postural adjustments, including the vestibular cortex and cerebellum, and promotes multisensory integration and neuroplasticity (31, 56). The highly immersive environment allows older adults to repeatedly expose themselves to challenging balance tasks in a safe virtual setting, thereby gradually reducing excessive fear of falling through error learning and positive reinforcement (57, 58). However, this study found that IVR had a relatively low SUCRA ranking across dynamic balance tests, suggesting that IVR may have limited advantages in terms of improving dynamic mobility. This may be because IVR training mainly focuses on standing postural control tasks and thus involves fewer dynamic activities such as walking and turning (59). For older adults whose primary goal is to improve dynamic mobility, IVR alone may thus not be sufficient to achieve optimal effects. Future studies could explore the synergistic effects of combining immersive and nonimmersive VR for improving dynamic balance, however.

Wii exergames demonstrated stable therapeutic effects on both static and dynamic balance, suggesting that the traditional exergames represented by Wii, while relatively simple, can improve balance function in older adults by using engagement to maintain training adherence and using real-time center-of-pressure feedback to guide older adults in the completion of weight-shifting and body coordination tasks in a standing posture (60). However, Wii had a relatively low SUCRA ranking regarding improving fear of falling, which may be related to its insufficient immersion and lack of targeted intervention content targeting fall-related psychological concerns (20). A previous meta-analysis in frail older adults did also show that Wii-based exergames were superior to conventional physical activity in improving TUG performance (61), which is consistent with the findings of the present study. In resource-constrained community or home-based settings, low-cost nonimmersive devices such as Wii may thus serve as effective alternatives (62).

BTSNVR training equipment achieved the highest SUCRA value (87.5%) in terms of improving dynamic balance outcome measures, significantly outperforming all other VR interventions. The BTS NIRVANA system is a projection-based interactive VR platform that creates virtual interactive scenes through floor or wall projections, thereby allowing patients to perform gait training, weight-shifting, and obstacle avoidance tasks within the projection area (63, 64). Its advantage lies in the fact that it integrates multisensory stimulation while providing simultaneous visual, auditory, and tactile feedback to enhance sensorimotor coupling (45). In addition, the training scenarios used in BTSNVR have a high degree of task specificity that matches the motor demands of the TUG test. It offers a variety of training modes for dynamic balance and gait, including movements such as turning, obstacle circumvention, and variable-speed walking, which are also required in the TUG test. Its training effects may thus be more consistent with dynamic mobility tasks in daily life as compared with those of other VR interventions (65). Yeşilyaprak et al. (45) similarly reported that BTSNVR training can be used to improve balance function and reduce fall risk in older adults.

This study also distinguished between AC and UC. The results showed that AC was significantly superior to UC in dynamic balance tests, yet displayed no significant advantages in terms of static balance or fear of falling. This finding suggests that AC can improve dynamic balance; however, unlike VR interventions, AC cannot specifically improve postural control and balance confidence through multisensory feedback and task-specific training. This result indirectly verifies the unique value of VR interventions in balance rehabilitation, which not only improve motor function but also promote sensory integration and neuroplasticity through cognitive-motor dual-task training (66). In addition, BioRescue demonstrated a relatively low SUCRA ranking in this study (33.5% for TUG), which may be related to the limited number of studies and insufficient sample size for BioRescue. Further studies are thus required to validate its efficacy.

This study has several limitations. First, the included primary studies exhibited considerable heterogeneity in terms of intervention duration, training frequency, session length, and specific VR game content. While a random-effects model was used to reduce the impact of heterogeneity on the pooled results, differences in intervention parameters across studies and clinical differences within the broad, platform-based intervention categories, which were retained because the limited number of RCTs made further subdivision unreliable, may still have affected the stability of the SUCRA ranking results. The SUCRA rankings in this study should therefore be interpreted cautiously as defining probabilistic optimality rather than definitive optimality. Second, some comparisons in this network meta-analysis, for example those between BTSNVR and IVR and between BioRescue and other interventions, relied on indirect evidence, as sufficient head-to-head randomized controlled trials are currently lacking. Future head-to-head trials directly comparing different VR interventions are thus needed to validate the current findings. Third, most included primary studies had small sample sizes and short follow-up periods, limiting reliable assessment of long-term effects. The overall validity was further limited by risk of bias. Specifically, 94.7% of studies were rated as having some concerns, and only one was rated as low risk. Key concerns included lack of participant and assessor blinding and selective reporting. These issues reduce confidence in the pooled estimates and should be considered a major limitation. Finally, this study did not conduct subgroup analyses based on different cognitive levels, for example by comparing effects in those with mild cognitive impairment with those in cognitively normal older adults, or by considering fall history, severity of balance impairment, or sex. These individual factors may moderate the efficacy of VR interventions, and thus more high-quality clinical studies need to be included in future research to explore these issues.

Despite its limitations, this study still makes several important contributions. It was the first to use a network meta-analysis approach to systematically compare the differences in efficacy of IVR, Kinect and Wii, a projection-based interactive VR system (BTSNVR), active control, and usual care, on balance function and dynamic mobility in older adults, thereby providing evidence-based medical evidence for the clinical application of different VR rehabilitation devices. Additionally, this study comprehensively assessed multidimensional clinical outcomes, including static balance, dynamic balance, and fear of falling, thereby offering a more comprehensive and clinically informative evidence map. The SUCRA ranking results of this study are preliminary. For older adults whose primary goal is to improve static balance and reduce fear of falling, Kinect or IVR may be the more advantageous choices, while for older adults whose primary goal is to improve dynamic balance and gait strategies, BTSNVR offers unique task-specific advantages, and Wii may also be an effective option. More generally, in resource-constrained community or home-based settings, low-cost nonimmersive devices such as Kinect and Wii offer good accessibility and potential for widespread promotion. The main contribution of this study is thus to provide evidence on the comparative efficacy of various VR interventions with respect to improving balance function in older adults, thereby offering a scientific basis for the selection and implementation of VR balance training devices in the target population.

## Data Availability

The original contributions presented in the study are included in the article/Supplementary material, further inquiries can be directed to the corresponding author.

## References

[1] James SL Lucchesi LR Bisignano C Castle CD Dingels ZV Fox JT . The global burden of falls: global, regional and national estimates of morbidity and mortality from the global burden of disease study 2017. Inj Prev. (2020) 26:i3–i11. doi: 10.1136/injuryprev-2019-043286 PMC7571347 31941758

[2] Kakara R Bergen G Burns E Stevens M. Nonfatal and fatal falls among adults aged ≥65 years - United States, 2020-2021. MMWR Morb Mortal Wkly Rep. (2023) 72:938–43. doi: 10.15585/mmwr.mm7235a1 37651272

[3] Xu D Wang Y Zhu S Zhao M Wang K. Relationship between fear of falling and quality of life in nursing home residents: the role of activity restriction. Geriatr Nurs. (2024) 57:45–50. doi: 10.1016/j.gerinurse.2024.03.006, 38520817

[4] Montero-Odasso M van der Velde N Martin FC Petrovic M Tan MP Ryg J . World guidelines for falls prevention and management for older adults: a global initiative. Age Ageing. (2022) 51:205. doi: 10.1093/ageing/afac205 PMC9523684 36178003

[5] Li Y Hou L Zhao H Xie R Yi Y Ding X. Risk factors for falls among community-dwelling older adults: a systematic review and Meta-analysis. Front Med (Lausanne). (2022) 9:1019094. doi: 10.3389/fmed.2022.1019094, 36687461 PMC9853191

[6] Xing L Bao Y Wang B Shi M Wei Y Huang X . Falls caused by balance disorders in the elderly with multiple systems involved: pathogenic mechanisms and treatment strategies. Front Neurol. (2023) 14:1128092. doi: 10.3389/fneur.2023.1128092, 36908603 PMC9996061

[7] Adjetey C Karnon B Falck RS Balasubramaniam H Buschert K Davis JC. Cost-effectiveness of exercise versus multimodal interventions that include exercise to prevent falls among community-dwelling older adults: a systematic review and Meta-analysis. Maturitas. (2023) 169:16–31. doi: 10.1016/j.maturitas.2022.12.003, 36630860

[8] Tiedemann A Sturnieks DL Burton E Thom JM Lord SR Scott S . Exercise and sports science Australia updated position statement on exercise for preventing falls in older people living in the community. J Sci Med Sport. (2025) 28:87–94. doi: 10.1016/j.jsams.2024.09.003 39341781

[9] Ratoza M Patel RM Brewer W Mitchell K Chevan J. Geographic disparities in rehabilitation provider availability and community demographics in Texas: a cross-sectional geographic information systems study. Phys Ther. (2026) 106:pzag014. doi: 10.1093/ptj/pzag014 41697017

[10] Atkins K Powell M Boyd M Cook L Ross S Bickel CS. Perceived challenges and solutions to providing rural physical therapy services: a qualitative inquiry of provider experience. Physiother Theory Pract. (2026) 42:710–24. doi: 10.1080/09593985.2025.2594571, 41319261

[11] Skolasky RL Colantuoni E Wegener ST Ali KJ McLaughlin KH. Arbor-telehealth study: an examination of telerehabilitation to improve function and reduce opioid use in persons with chronic low back pain in rural communities - protocol of a pragmatic, individually randomised group treatment trial. BMJ Open. (2025) 15:e102773. doi: 10.1136/bmjopen-2025-102773 PMC12161378 40499966

[12] Chen Y Shah S Chen Y Owen AJ Ekegren CL Ilic D . Barriers to and facilitators of physical activity among community-dwelling older adults: a systematic review. BMJ Open. (2025) 15:e095260. doi: 10.1136/bmjopen-2024-095260, 40854831 PMC12382567

[13] Kilgour AHM Rutherford M Higson J Meredith SJ McNiff J Mitchell S . Barriers and motivators to undertaking physical activity in adults over 70-a systematic review of the quantitative literature. Age Ageing. (2024) 53:fae080. doi: 10.1093/ageing/afae080 PMC11036106 38651329

[14] Machacova K Polanska H Steffl M Bartova A Novotna B Holmerova I. Revolution or empty promise? A 6- and 12-month follow-up to an RCT of the online exercise program Permanento in older adults. Clin Interv Aging. (2025) 20:1519–35. doi: 10.2147/cia.S530709 PMC12433213 40950777

[15] Høeg ER Povlsen TM Bruun-Pedersen JR Lange B Nilsson NC Haugaard KB . System immersion in virtual reality-based rehabilitation of motor function in older adults: a systematic review and meta-analysis. Front Virtual Real. (2021) 2:647993. doi: 10.3389/frvir.2021.647993

[16] Qin R Li Z Huang K Luan T Miao B Gong L . Comparative efficacy of various of virtual reality technologies on cognitive function in older adults with mild cognitive impairment: a systematic review and network Meta-analysis. J Am Med Dir Assoc. (2025) 26:105941. doi: 10.1016/j.jamda.2025.105941, 41109334

[17] Park JH Jeon HS Kim JH Kim YJ Moon GA. Effectiveness of non-immersive virtual reality exercises for balance and gait improvement in older adults: a Meta-analysis. Technol Health Care. (2024) 32:1223–38. doi: 10.3233/thc-230156, 37781824

[18] Lee J Phu S Lord SR Okubo Y. Effects of immersive virtual reality training on balance, gait and mobility in older adults: a systematic review and Meta-analysis. Gait Posture. (2024) 110:129–37. doi: 10.1016/j.gaitpost.2024.03.009, 38581933

[19] Cieślik B Wrzeciono A Mazurek J Federico S Szczepańska-Gieracha J Kiper P. Balance board or motion capture? A meta-analysis exploring the effectiveness of commercially available virtual reality exergaming in enhancing balance and functional mobility among the elderly. Games Health J. (2024) 13:407–18. doi: 10.1089/g4h.2023.0109 39093844

[20] Liu H Xing Y Wu Y. Effect of Wii fit exercise with balance and lower limb muscle strength in older adults: a Meta-analysis. Front Med (Lausanne). (2022) 9:812570. doi: 10.3389/fmed.2022.812570, 35602499 PMC9120538

[21] Cieślik B Mazurek J Wrzeciono A Maistrello L Szczepańska-Gieracha J Conte P . Examining technology-assisted rehabilitation for older adults' functional mobility: a network Meta-analysis on efficacy and acceptability. NPJ Digit Med. (2023) 6:159. doi: 10.1038/s41746-023-00907-7, 37620411 PMC10449892

[22] Hutton B Salanti G Caldwell DM Chaimani A Schmid CH Cameron C . The Prisma extension statement for reporting of systematic reviews incorporating network Meta-analyses of health care interventions: checklist and explanations. Ann Intern Med. (2015) 162:777–84. doi: 10.7326/m14-2385, 26030634

[23] Nikolakopoulou A Higgins JPT Papakonstantinou T Chaimani A Del Giovane C Egger M . Cinema: an approach for assessing confidence in the results of a network meta-analysis. PLoS Med. (2020) 17:e1003082. doi: 10.1371/journal.pmed.1003082 PMC7122720 32243458

[24] Mansournia MA Collins GS Nielsen RO Nazemipour M Jewell NP Altman DG . A checklist for statistical assessment of medical papers (the champ statement): explanation and elaboration. Br J Sports Med. (2021) 55:1009–17. doi: 10.1136/bjsports-2020-103652, 33514558 PMC9110112

[25] Higgins JP Jackson D Barrett JK Lu G Ades AE White IR. Consistency and inconsistency in network Meta-analysis: concepts and models for multi-arm studies. Res Synth Methods. (2012) 3:98–110. doi: 10.1002/jrsm.1044, 26062084 PMC4433772

[26] White IR Barrett JK Jackson D Higgins JP. Consistency and inconsistency in network Meta-analysis: model estimation using multivariate Meta-regression. Res Synth Methods. (2012) 3:111–25. doi: 10.1002/jrsm.1045, 26062085 PMC4433771

[27] Veroniki AA Vasiliadis HS Higgins JP Salanti G. Evaluation of inconsistency in networks of interventions. Int J Epidemiol. (2013) 42:332–45. doi: 10.1093/ije/dys222 PMC5411010 23508418

[28] Doosti-Irani A Nazemipour M Mansournia MA. What are network Meta-analyses (Nmas)? A primer with four tips for clinicians who read Nmas and who perform them (methods matter series). Br J Sports Med. (2020). doi: 10.1136/bjsports-2020-102872, 32948517

[29] Chaimani A Higgins JP Mavridis D Spyridonos P Salanti G. Graphical tools for network meta-analysis in Stata. PLoS One. (2013) 8:e76654. doi: 10.1371/journal.pone.0076654 PMC3789683 24098547

[30] Yousefi Babadi S Daneshmandi H. Effects of virtual reality versus conventional balance training on balance of the elderly. Exp Gerontol. (2021) 153:111498. doi: 10.1016/j.exger.2021.111498, 34311059

[31] Campo-Prieto P Cancela-Carral JM Rodríguez-Fuentes G. Feasibility and effects of an immersive virtual reality exergame program on physical functions in institutionalized older adults: a randomized clinical trial. Sensors (Basel). (2022) 22:6742. doi: 10.3390/s22186742 PMC9505598 36146092

[32] Delbroek T Vermeylen W Spildooren J. The effect of cognitive-motor dual task training with the Biorescue force platform on cognition, balance and dual task performance in institutionalized older adults: a randomized controlled trial. J Phys Ther Sci. (2017) 29:1137–43. doi: 10.1589/jpts.29.1137, 28744033 PMC5509577

[33] Fakhro MA Hadchiti R Awad B. Effects of Nintendo Wii fit game training on balance among Lebanese older adults. Aging Clin Exp Res. (2020) 32:2271–8. doi: 10.1007/s40520-019-01425-x, 31802405

[34] Gomes GCV Simões MDS Lin SM Bacha JMR Viveiro LAP Varise EM . Feasibility, safety, acceptability, and functional outcomes of playing Nintendo Wii fit plus(tm) for frail older adults: a randomized feasibility clinical trial. Maturitas. (2018) 118:20–8. doi: 10.1016/j.maturitas.2018.10.002 30415751

[35] Jung DI Ko DS Jeong MA. Kinematic effect of Nintendo Wii(tm) sports program exercise on obstacle gait in elderly women with falling risk. J Phys Ther Sci. (2015) 27:1397–400. doi: 10.1589/jpts.27.1397, 26157228 PMC4483406

[36] Kwok BC Pua YH. Effects of Wiiactive exercises on fear of falling and functional outcomes in community-dwelling older adults: a randomised control trial. Age Ageing. (2016) 45:621–7. doi: 10.1093/ageing/afw108, 27496921

[37] Lee M Son J Kim J Yoon B. Individualized feedback-based virtual reality exercise improves older women's self-perceived health: a randomized controlled trial. Arch Gerontol Geriatr. (2015) 61:154–60. doi: 10.1016/j.archger.2015.06.010, 26145489

[38] Liao YY Chen IH Wang RY. Effects of Kinect-based exergaming on frailty status and physical performance in Prefrail and frail elderly: a randomized controlled trial. Sci Rep. (2019) 9:9353. doi: 10.1038/s41598-019-45767-y, 31249332 PMC6597702

[39] Merriman NA Whyatt C Setti A Craig C Newell FN. Successful balance training is associated with improved multisensory function in fall-prone older adults. Comput Human Behav. (2015) 45:192–203. doi: 10.1016/j.chb.2014.12.017

[40] Molhemi F Monjezi S Mehravar M Shaterzadeh-Yazdi MJ Salehi R Hesam S . Effects of virtual reality vs conventional balance training on balance and falls in people with multiple sclerosis: a randomized controlled trial. Arch Phys Med Rehabil. (2021) 102:290–9. doi: 10.1016/j.apmr.2020.09.395, 33161005

[41] Montero-Alía P Miralles-Basseda R López-Jiménez T Muñoz-Ortiz L Jiménez-González M Prat-Rovira J . Controlled trial of balance training using a video game console in community-dwelling older adults. Age Ageing. (2019) 48:506–12. doi: 10.1093/ageing/afz047, 31081504 PMC6593319

[42] Padala KP Padala PR Malloy TR Geske JA Dubbert PM Dennis RA . Wii-fit for improving gait and balance in an assisted living facility: a pilot study. J Aging Res. (2012) 2012:597573. doi: 10.1155/2012/597573 PMC3382377 22745909

[43] Stanmore EK Mavroeidi A de Jong LD Skelton DA Sutton CJ Benedetto V . The effectiveness and cost-effectiveness of strength and balance exergames to reduce falls risk for people aged 55 years and older in UK assisted living facilities: a multi-Centre, cluster randomised controlled trial. BMC Med. (2019) 17:49. doi: 10.1186/s12916-019-1278-9 PMC6394073 30813926

[44] Tsang WW Fu AS. Virtual reality exercise to improve balance control in older adults at risk of falling. Hong Kong Med J. (2016) 22:S19–22. 26908338

[45] Yeşilyaprak SS Yıldırım M Tomruk M Ertekin Ö Algun ZC. Comparison of the effects of virtual reality-based balance exercises and conventional exercises on balance and fall risk in older adults living in nursing homes in Turkey. Physiother Theory Pract. (2016) 32:191–201. doi: 10.3109/09593985.2015.1138009, 27049879

[46] Yoo HN Chung E Lee BH. The effects of augmented reality-based Otago exercise on balance, gait, and falls efficacy of elderly women. J Phys Ther Sci. (2013) 25:797–801. doi: 10.1589/jpts.25.797, 24259856 PMC3820389

[47] Zahedian-Nasab N Jaberi A Shirazi F Kavousipor S. Effect of virtual reality exercises on balance and fall in elderly people with fall risk: a randomized controlled trial. BMC Geriatr. (2021) 21:509. doi: 10.1186/s12877-021-02462-w, 34563120 PMC8465759

[48] Parmak D Angın E Iyigun G. Effects of immersive virtual reality on physical function, fall-related outcomes, fatigue, and quality of life in older adults: a randomized controlled trial. Healthcare (Basel). (2025) 13:1800. doi: 10.3390/healthcare13151800 PMC12346243 40805833

[49] Yang CM Chen Hsieh JS Chen YC Yang SY Lin HK. Effects of Kinect exergames on balance training among community older adults: a randomized controlled trial. Medicine (Baltimore). (2020) 99:e21228. doi: 10.1097/md.0000000000021228 PMC7360267 32664177

[50] Bacha JMR Gomes GCV de Freitas TB Viveiro LAP da Silva KG Bueno GC . Effects of Kinect adventures games versus conventional physical therapy on postural control in elderly people: a randomized controlled trial. Games Health J. (2018) 7:24–36. doi: 10.1089/g4h.2017.0065, 29239677

[51] Freed SA Sprague BN Stephan AT Doyle CE Tian J Phillips CB . Feasibility and enjoyment of exercise video games in older adults. Front Public Health. (2021) 9:751289. doi: 10.3389/fpubh.2021.751289, 34805074 PMC8602072

[52] Sheehy L Bharadwaj L Nissen KA Estey JL. Non-immersive virtual reality exercise can increase exercise in older adults living in the community and in long-term care: a randomized controlled trial. Clin Interv Aging. (2025) 20:109–24. doi: 10.2147/cia.S498272, 39931102 PMC11807768

[53] Singh DK Rajaratnam BS Palaniswamy V Raman VP Bong PS Pearson H. Effects of balance-focused interactive games compared to therapeutic balance classes for older women. Climacteric. (2013) 16:141–6. doi: 10.3109/13697137.2012.664832, 22640573

[54] Fidan O Kiloatar H Colak E Oskay D. Effects of group-based virtual reality training on activities of daily living and functional outcomes in older adults: a randomised control trial. Disabil Rehabil Assist Technol. (2025) 20:1044–55. doi: 10.1080/17483107.2024.2431051, 39570714

[55] Kim SH Cho SH. Benefits of virtual reality program and motor imagery training on balance and fall efficacy in isolated older adults: a randomized controlled trial. Medicina (Kaunas). (2022) 58:1545. doi: 10.3390/medicina58111545, 36363502 PMC9692723

[56] Yue T Yan X Lv Y Ren X Zhang S Qi F. Effects of immersive virtual reality-based tennis training on balance in older adults. Gerontology. (2025) 71:861–71. doi: 10.1159/000547548, 40695260

[57] Campo-Prieto P Cancela-Carral JM Alsina-Rey B Rodríguez-Fuentes G. Immersive virtual reality as a novel physical therapy approach for nonagenarians: usability and effects on balance outcomes of a game-based exercise program. J Clin Med. (2022) 11:3911. doi: 10.3390/jcm11133911, 35807196 PMC9267554

[58] Trillo-Charlín I Bravo-Aparicio J Avendaño-Coy J Beltrán-Alacreu H. Impact of 4 weeks or more immersive virtual reality on quality of life and physical activity in older adults: systematic review and meta-analysis. JMIR Aging. (2026) 9:e80820. doi: 10.2196/80820, 41525705 PMC12795489

[59] Lima Rebêlo F de Souza Silva LF Doná F Sales Barreto A de Souza Siqueira Quintans J. Immersive virtual reality is effective in the rehabilitation of older adults with balance disorders: a randomized clinical trial. Exp Gerontol. (2021) 149:111308. doi: 10.1016/j.exger.2021.111308, 33744393

[60] Rendon AA Lohman EB Thorpe D Johnson EG Medina E Bradley B. The effect of virtual reality gaming on dynamic balance in older adults. Age Ageing. (2012) 41:549–52. doi: 10.1093/ageing/afs053, 22672915

[61] Alhasan H Alandijani E Bahamdan L Khudary G Aburaya Y Awali A . Home-based virtual reality training for enhanced balance, strength, and mobility among older adults with frailty: systematic review and Meta-analysis. JMIR Serious Games. (2025) 13:e67146. doi: 10.2196/67146, 40680186 PMC12294645

[62] Carcelén-Fraile MDC Aibar-Almazán A Hita-Contreras F Sánchez-Alcalá M Parra-Díaz AB Infante-Guedes A . Using the Nintendo™ Wii to improve physical function and reduce the risk of falls in older adults: a randomized controlled clinical trial. Sensors (Basel). (2024) 24:6358. doi: 10.3390/s24196358, 39409396 PMC11478887

[63] Park BM Choi H Jeong H. Effects of virtual reality based on fall prevention intervention: a systematic review and Meta-analysis. Healthcare (Basel). (2025) 13:1845. doi: 10.3390/healthcare13151845, 40805878 PMC12346508

[64] De Luca R Russo M Naro A Tomasello P Leonardi S Santamaria F . Effects of virtual reality-based training with Bts-Nirvana on functional recovery in stroke patients: preliminary considerations. Int J Neurosci. (2018) 128:791–6. doi: 10.1080/00207454.2017.1403915 29148855

[65] Nightingale CJ Mitchell SN Butterfield SA. Validation of the timed up and go test for assessing balance variables in adults aged 65 and older. J Aging Phys Act. (2019) 27:230–3. doi: 10.1123/japa.2018-0049, 30117359

[66] Schaumburg M Imtiaz A Zhou R Bernard M Wolbers T Segen V. Immersive virtual reality for older adults: challenges and solutions in basic research and clinical applications. Ageing Res Rev. (2025) 109:102771. doi: 10.1016/j.arr.2025.102771, 40373984

