# Supplementary material for: Comparative efficacy of different virtual reality-based interventions for static balance, dynamic balance, and fear of falling in older adults: a systematic review and network meta-analysis
Source: Front Public Health. 2026 Oct 20;14:1973306. doi: 10.3389/fpubh.2026.1973306 (PMC13727423; doi:10.3389/fpubh.2026.1973306)
Supplement: Supplementary file 1 [file Data_Sheet_1.docx]

| Table S1. Pubmed Search Strategy. | |  |
| --- | --- | --- |
| # | Search terms | Results |
| 1 | "Aged"[Mesh] OR "aged"[tiab] OR "elderly"[tiab] OR "older adult"[tiab] OR "older adults"[tiab] OR "geriatric"[tiab] OR "community-dwelling"[tiab] OR "nursing home"[tiab] OR "frail elderly"[tiab] | 4,690,481 |
| 2 | "Virtual Reality"[Mesh] OR "virtual reality"[tiab] OR "augmented reality"[tiab] OR "exergame"[tiab] OR "exergames"[tiab] OR "exergaming"[tiab] OR "active video game"[tiab] OR "active video games"[tiab] OR "Nintendo"[tiab] OR "Wii"[tiab] OR "Kinect"[tiab] OR "Xbox"[tiab] OR "Oculus"[tiab] OR "Meta Quest"[tiab] OR "head-mounted display"[tiab] OR "HMD"[tiab] OR "immersive virtual reality"[tiab] OR "non-immersive virtual reality"[tiab] OR "interactive video game"[tiab] OR "BTS NIRVANA"[tiab] OR "MIRA Rehab"[tiab] | 43,933 |
| 3 | "Postural Balance"[Mesh] OR "balance"[tiab] OR "Berg Balance Scale"[tiab] OR "BBS"[tiab] OR "Timed Up and Go"[tiab] OR "TUG"[tiab] OR "Falls Efficacy Scale"[tiab] OR "FES"[tiab] OR "FES-I"[tiab] OR "fear of falling"[tiab] OR "fall risk"[tiab] | 404771 |
| 4 | "Randomized Controlled Trial"[Publication Type] OR "randomized"[tiab] OR "randomised"[tiab] OR "RCT"[tiab] | 1,223,706 |
| 5 | #1 AND #2 AND #3 AND #4 | 484 |

| Table S2. Intervention details of the included studies | |
| --- | --- |
| Study | Intervention Details |
| Babadi  & Daneshmandi,2021 | Kinect-based VR training included boxing, table tennis, soccer, golf, ski slalom and American football. |
| Campo-Prieto et al.,2022 | Immersive VR training based on BOX VR exergame included hitting colored balls, uppercut/cross/hook boxing movements, blocking with both hands, and trunk/limb shifting. |
| Delbroek et al., 2017 | BioRescue VR training including memory card matching, obstacle avoidance walking, hot air balloon, blackboard, spaceshuttle, maze, tortoise, rally, and downhill skiing, all performed via weight‑shift control. |
| Fakhro et al., 2019 | Wii-based VR training included two Wii Fit games (soccer heading and table tilt), both performed via weight‑shift control on the balance board. |
| Gomes et al., 2018 | Wii-based VR training included table tilt, rhythm parade, obstacle course, single leg extension, tilt city, basic step, penguin slide, heading soccer, basic run plus, and torso twist, all controlled via weight‑shifting on the wii balance board. |
| Jung et al., 2015 | Wii-based VR training included wakeboard, frisbee dog, jet ski, and canoe games, all controlled by swinging, rowing, or tilting the remote controllers |
| Kwok et al., 2016 | Wii-based VR training included cardiovascular training, resistance band strengthening, calisthenics and balance training. |
| Lee et al., 2015 | Kinect-based VR training included bilateral arm abduction with single leg lift, unilateral elbow flexion with ipsilateral knee flexion, double knee flexion with shoulder-level arm abduction, cross-arm standing knee flexion movements. |
| Merriman et al., 2015 | Wii-based VR training included apple‑catching (shifting balance to control a basket) and bubble‑popping (shifting balance to move a character in a dynamic sea‑scape). |
| Molhemi et al., 2020 | Kinect-based VR training included “Light Race” (stepping forward/backward/left/right to turn off randomly lit virtual lights), “Stack'em up” (using a virtual stick to collect boxes falling from the top and placing them in specific locations), and “20,000 Leaks” (shifting weight, reaching, and stepping to prevent leaks from random breaking parts on aquarium walls and floor) |
| Montero-Alía et al., 2019 | Wii-based VR training included balance bubble, soccer heading, ski jump, table tilt, ski slalom, penguin slide, snowboard slalom, and tightrope walk. |
| Padala et al., 2012 | Wii-based VR training included yoga poses, strength exercises and balance games; yoga: halfmoon, warrior pose, chair, sun salutation; strength: single leg extensions, lunges, torso twists; balance: soccer heading, ski slalom, ski jump, table tilt, balance bubble, penguin slide |
| Parmak et al., 2025 | Immersive VR training using Oculus Meta Quest 2 headset with FIT‑XR application included Box mode (hitting blue and yellow balls with corresponding colored gloves, avoiding obstacles requiring lateral movement or squatting) and Slam mode (moving around a 9 m² area to hit blue, yellow, and pink balls with designated hands before time expires). |
| Stanmore et al., 2019 | Kinect-based VR training included strength and balance gamified tasks adapted from otago and fame exercise programmes, with personalised progressive game modules targeting lower limb strength, static and dynamic balance, and weight shifting movements |
| Tsang & Fu, 2016 | Wii-based VR training included soccer heading, table tilt and balance bubble games |
| Yeşilyaprak et al., 2016 | BTS NIRVANA VR Interactive System balance training including: warm‑up (Water Classic: stepping on waving water; Dew 100: walking and wall sliding), training games (Motion & Water Classic: stability and dynamic activity; Hearts: static balance and stabilization; Sprites: functional reach and dynamic control; Butterfly: static and dynamic balance; Knee Up: lifting knees while walking; Tap the Mole: upper/lower extremity and trunk dynamic training for balance and reaction time), and cool‑down (Dew 100 and Water Classic). |
| Yoo et al., 2013 | Immersive VR training included knee extension, knee flexion, hip abduction, plantar flexion, dorsiflexion, backward walking, turning, heel-to-toe walking, single leg stand, heel walk, toe walk, backward tandem walk, sit-to-stand and stair stepping tasks |
| Zahedian‑Nasab et al., 2021 | Kinect-based VR training included ski, penalty and goalkeeper soccer, and darts interactive balance tasks. |

Figure S1. RoB of included studies

| Table S3. Study characteristics relevant to the assessment of transitivity | | | | | | | |
| --- | --- | --- | --- | --- | --- | --- | --- |
| Study | Residential or care setting and study population | Female-to-male ratio | Age  (Mean ± SD) | Intervention approach (stand-alone/add-on) | Intervention  (Duration, Frequency and Cycle) | Baseline cognitive status | Baseline balance function |
| Babadi & Daneshmandi, 2021 | Nursing home residents | 19∶17 | Kinect：66.5±3.8  AC：67.5±3.11  UC：66.75±3.27 | Stand-alone | Kinect-based VR training,  60 min/session, 3 times/week for 9 weeks | All participants had MMSE scores >24. | Able to stand independently for 90 seconds. |
| Campo-Prieto et al., 2022 | Institutionalized older adults | 7∶1 | IVR: 85.08±8.48  UC: 84.82±8.10 | Add-on | Immersive VR training,  6 min/session, 3 times/week for 10 weeks | Individuals with cognitive impairment preventing comprehension of the intervention were excluded. | Able to stand without assistance. |
| Delbroek et al., 2017 | Nursing home residents | 13∶7 | BioRescue: 86.9 ± 5.6  UC :  87.5 ± 6.6 | Add-on | BioRescue VR training,  18–30 min/session, 2 times/week for 6 weeks | Mild cognitive impairment (MoCA <26); individuals with diagnosed dementia were excluded. | Able to walk 10 m repeatedly. |
| Fakhro et al., 2019 | Institutionalized older adults | Matched by sex | Total:  72.2 ± 5.2 | Stand-alone | Wii-based VR training,  40 min/session, 3 times/week for 8 weeks | All participants had MoCA≥23. | Impaired dynamic balance; TUG >14 seconds was an inclusion criterion. |
| Gomes et al., 2018 | Recruited from outpatient clinics | 14∶1 | Wii：  83 ± 5.87  UC:  85 ± 6.19 | Stand-alone | Wii-based VR training,  50 min/session, 2 sessions /week for 7 weeks | Individuals with cognitive difficulties that would interfere with Wii use were excluded. | Able to walk independently and exercise in a standing position. |
| Jung et al., 2015 | Not clearly specified | 24∶0 | Wii: 74.3 ± 2.1  AC: 74.3 ± 3.5  UC: 73.6 ± 2.4 | Stand-alone | Wii-based VR training,  30 min/session, 2 sessions /week for 8 weeks | Not reported. | Tinetti-POMA  score: 19–24. |
| Kwok et al., 2016 | Community-dwelling older adults with mild to moderate physical frailty | 17∶3 | Wii: 70.5±6.7  AC: 69.8±7.5 | Add-on | Wii-based VR training,  60 min/session, 1 session/week for 12 weeks | AMT (maximum score: 10): Wii group, 8.9 ± 1.1; gym group, 8.9 ± 1.3. | SPPB 5～9、MFES≤9 |
| Lee et al., 2015 | Not clearly specified | 100% female | Kinect：68.77±4.62  AC: 67.71±4.31 | Stand-alone | Kinect-based VR training,  60 min/session, 3 times/week, for 8 weeks | All participants had MMSE scores >23. | Able to stand independently. |
| Liao et al., 2019 | Prefrail and frail older adults recruited from day care centers | 9∶4 | Kinect：79.6±8.5  AC:  84.1±5.5 | Add-on | Kinect-based VR training,  60 min/session, 3 sessions/week for 12 weeks | Not reported. | Baseline TUG: VR group, 17.0 ± 8.4 s; control group, 16.6 ± 9.9 s |
| Merriman et al., 2015 | Community-dwelling participants and residents of sheltered housing for retired people | 15：4 | Total：74  (range ≥60) | Stand-alone | Wii-based VR training,  30 min/session, 2 sessions/week for 5 weeks | All participants  had MMSE scores >24. | Baseline BBS:  VR group, 50.24 ± 6.57; control  group, 48.84 ± 9.95. |
| Molhemi et al., 2020 | Not clearly specified | 8：5 | Kinect：  66.8 ± 8.4 AC :  67.6 ± 8.4 | Stand-alone | Kinect-based VR training,  30 min/session, 3 sessions/week for 6 weeks | All participants had MMSE scores >24. | Baseline BBS: VR group, 46.6 ± 3.9; control group, 45.5 ± 7.2. |
| Montero‑Alía et al., 2019 | Community-dwelling older adults | 577：400 | Median age:75.3 | Stand-alone | Wii-based VR training,  30 min/session, 2 sessions/week for 12 weeks | No cognitive impairment: 902 participants (96.6%); mild cognitive impairment: 32 participants (3.4%). | Able to walk independently. |
| Padala et al., 2012 | Residents of assisted living facilities | 8：3 |  | Stand-alone | Wii-based VR training,  30 min/session, 5 sessions/week for 8 weeks | Mild Alzheimer’s dementia. | High fall risk; mean baseline BBS scores were below 45 in both groups: Wii group, 43.4 ± 8.9; walking group, 41.3 ± 7.6. |
| Parmak et al., 2025 | Community-dwelling older adults | 36：8 | IVR:  71.14 ± 4.83 AC:  75.36 ± 9.16 | Stand-alone | Immersive VR training,  35 min/session, 3 sessions/week for 8 weeks | MMST ≥24. | Baseline FAB: IVR group, 28.77 ± 4.68; control group, 27.32 ± 6.59. |
| Stanmore et al., 2019 | Residents of assisted living facilities | 83：23 | Kinect：  77.9 ± 8.9  AC:  77.8 ± 10.2 | Add-on | Kinect-based VR training,  3 sessions/week for 12 weeks | Able to understand exercise and game instructions. | Baseline BBS: intervention group, 42.0 ± 13.5; control group, 40.6 ± 13.1. |
| Tsang & Fu, 2016 | Nursing home residents | 48：31 | Wii: 82.3±3.8  AC: 82.0±4.3 | Stand-alone | Wii-based VR training,  60 min/session, 3 sessions/week for 6 weeks | Not reported. | Older adults with a history of falls and limited walking ability |
| Yeşilyaprak et al., 2016 | Nursing home residents | 12：6 | BTSNVR: 70.1 ± 4.0 AC:  73.1 ± 4.5 | Stand-alone | BTS NIRVANA VR training, 40–50 min/session,  3 sessions/week for 6 weeks | MMSE ≥21. | Baseline BBS: VR group, 51.0 ± 2.7; conventional training group, 49.6 ± 3.1. |
| Yoo et al., 2013 | Not clearly specified | 100%  female | IVR:  72.90 ± 3.41  AC:  75.64 ± 5.57 | Stand-alone | Immersive VR training,  60 min/session, 3 sessions/week for 12 weeks | MMSE≥24. | Baseline BBS: IVR group, 47.60 ± 5.36; Otago exercise group, 48.91 ± 4.53. |
| Zahedian-Nasab et al., 2021 | Nursing home residents | 16：44 | Kinect：69.67±7.72  AC: 72.00±7.81 | Stand-alone | Kinect-based VR training,  30–60 min/session,  2 sessions/week for 6 weeks | Individuals with a history of cognitive, psychiatric, or physical conditions that could interfere with exercise were excluded. | Balance impairment; TUG of 14–20 seconds was an inclusion criterion. |
| Babadi & Daneshmandi, 2021 | Nursing home residents | 19∶17 | Kinect：66.5±3.8  AC：67.5±3.11  UC：66.75±3.27 | Stand-alone | Kinect-based VR training,  60 min/session, 3 times/week for 9 weeks | All participants had MMSE scores >24. | Able to stand independently for 90 seconds. |
| Abbreviations: AC, active control; UC, usual care; VR, virtual reality; IVR, immersive virtual reality; AR, augmented reality; BTSNVR, BTS NIRVANA virtual reality; SD, standard deviation; MMSE, Mini-Mental State Examination; MMST, Mini-Mental State Test; MoCA, Montreal Cognitive Assessment; AMT, Abbreviated Mental Test; BBS, Berg Balance Scale; FAB, Fullerton Advanced Balance Scale; POMA, Performance-Oriented Mobility Assessment; SPPB, Short Physical Performance Battery; MFES, Modified Falls Efficacy Scale; TUG, Timed Up and Go test. | | | | | | | |

Figure S2. Results of Loop-specific inconsistency test

| Table S4. League table of the static balance | | | | | |
| --- | --- | --- | --- | --- | --- |
| Kinect | -0.03  (-6.15,6.08) | -1.89  (-7.58,3.79) | -2.94  (-7.33,1.44) | -3.09  (-6.29,0.11) | -6.83  (-11.89,-1.78) |
| 0.03  (-6.08,6.15) | IVR | -1.86  (-8.88,5.16) | -2.91  (-8.93,3.10) | -3.06  (-8.27,2.15) | -6.80  (-13.33,-0.28) |
| 1.89  (-3.79,7.58) | 1.86  (-5.16,8.88) | BTSNVR | -1.05  (-6.63,4.53) | -1.20  (-5.90,3.50) | -4.94  (-11.07,1.18) |
| 2.94  (-1.44,7.33) | 2.91  (-3.10,8.93) | 1.05  (-4.53,6.63) | Wii | -0.15  (-3.15,2.85) | -3.89  (-7.44,-0.34) |
| 3.09  (-0.11,6.29) | 3.06  (-2.15,8.27) | 1.20  (-3.50,5.90) | 0.15  (-2.85,3.15) | AC | -3.74  (-7.67,0.19) |
| 6.83 (1.78,11.89) | 6.80 (0.28,13.33) | 4.94  (-1.18,11.07) | 3.89  (0.34,7.44) | 3.74  (-0.19,7.67) | UC |

| Table S5. League table of the dynamic balance | | | | | | |
| --- | --- | --- | --- | --- | --- | --- |
| BTSNVR | 0.57  (-1.67,2.80) | 1.49  (-0.64,3.61) | 1.70  (-0.34,3.74) | 3.96  (-6.48,14.40) | 3.36  (-0.82,7.54) | 3.36 (1.20,5.52) |
| -0.57  (-2.80,1.67) | Wii | 0.92  (-0.23,2.07) | 1.13 (0.22,2.04) | 3.39  (-6.89,13.67) | 2.79  (-0.96,6.55) | 2.79 (1.66,3.93) |
| -1.49  (-3.61,0.64) | -0.92  (-2.07,0.23) | Kinect | 0.21  (-0.38,0.81) | 2.47  (-7.76,12.71) | 1.87  (-1.76,5.51) | 1.87 (1.22,2.53) |
| -1.70  (-3.74,0.34) | -1.13  (-2.04,-0.22) | -0.21  (-0.81,0.38) | AC | 2.26  (-7.98,12.50) | 1.66  (-1.98,5.30) | 1.66 (0.97,2.35) |
| -3.96  (-14.40,6.48) | -3.39  (-13.67,6.89) | -2.47  (-12.71,7.76) | -2.26  (-12.50,7.98) | BioRescue | -0.60  (-11.42,10.22) | -0.60  (-10.82,9.62) |
| -3.36  (-7.54,0.82) | -2.79  (-6.55,0.96) | -1.87  (-5.51,1.76) | -1.66  (-5.30,1.98) | 0.60  (-10.22,11.42) | IVR | 0.00  (-3.58,3.58) |
| -3.36  (-5.52,-1.20) | -2.79  (-3.93,-1.66) | -1.87  (-2.53,-1.22) | -1.66  (-2.35,-0.97) | 0.60  (-9.62,10.82) | -0.00  (-3.58,3.58) | UC |

| Table S6. League table of the fear of falling | | | | | |
| --- | --- | --- | --- | --- | --- |
| Kinect | 0.31  (-4.87,5.50) | 0.65  (-5.34,6.65) | 1.35  (-1.72,4.43) | 2.95 (0.13,5.77) | 3.52 (1.56,5.48) |
| -0.31  (-5.50,4.87) | IVR | 0.34  (-6.29,6.97) | 1.04  (-3.14,5.22) | 2.63  (-3.26,8.53) | 3.21  (-2.33,8.75) |
| -0.65  (-6.65,5.34) | -0.34  (-6.97,6.29) | BTSNVR | 0.70  (-4.45,5.85) | 2.29  (-4.33,8.91) | 2.87  (-3.44,9.17) |
| -1.35  (-4.43,1.72) | -1.04  (-5.22,3.14) | -0.70  (-5.85,4.45) | AC | 1.59  (-2.57,5.75) | 2.17  (-1.47,5.80) |
| -2.95  (-5.77,-0.13) | -2.63  (-8.53,3.26) | -2.29  (-8.91,4.33) | -1.59  (-5.75,2.57) | Wii | 0.57  (-1.45,2.59) |
| -3.52  (-5.48,-1.56) | -3.21  (-8.75,2.33) | -2.87  (-9.17,3.44) | -2.17  (-5.80,1.47) | -0.57  (-2.59,1.45) | UC |

| Table S7. Confidence in the evidence for each treatment comparison for static balance | | | | | | | | |
| --- | --- | --- | --- | --- | --- | --- | --- | --- |
| Comparison | Number of studies | Within-study bias | Reporting bias | Indirectness | Imprecision | Heterogeneity | Incoherence | Confidence rating |
| Mixed evidence | | | | | | | | |
| AC:BTSNVR | 1 | Some concerns | Low risk | No concerns | Major concerns | No concerns | Major concerns | Very low |
| AC:IVR | 1 | Some concerns | Low risk | No concerns | Some concerns | Some concerns | Major concerns | Very low |
| AC:Kinect | 3 | Some concerns | Low risk | No concerns | Some concerns | Some concerns | Major concerns | Very low |
| AC:UC | 1 | Some concerns | Low risk | No concerns | Some concerns | Some concerns | No concerns | Low |
| AC:Wii | 3 | Some concerns | Low risk | No concerns | No concerns | Major concerns | No concerns | Low |
| UC:Wii | 2 | Some concerns | Low risk | No concerns | Some concerns | Some concerns | No concerns | Low |
| Indirect evidence | | | | | | | | |
| BTSNVR:IVR | 0 | Some concerns | Low risk | No concerns | Major concerns | No concerns | Major concerns | Very low |
| BTSNVR:Kinect | 0 | Some concerns | Low risk | No concerns | Major concerns | No concerns | Major concerns | Very low |
| BTSNVR:UC | 0 | Some concerns | Low risk | No concerns | Some concerns | Some concerns | Major concerns | Very low |
| BTSNVR:Wii | 0 | Some concerns | Low risk | No concerns | Major concerns | No concerns | Major concerns | Very low |
| IVR:Kinect | 0 | Some concerns | Low risk | No concerns | Major concerns | No concerns | Major concerns | Very low |
| IVR:UC | 0 | Some concerns | Low risk | No concerns | Some concerns | Some concerns | Major concerns | Very low |
| IVR:Wii | 0 | Some concerns | Low risk | No concerns | Major concerns | No concerns | Major concerns | Very low |
| Kinect:UC | 0 | Some concerns | Low risk | No concerns | No concerns | Major concerns | Major concerns | Very low |
| Kinect:Wii | 0 | Some concerns | Low risk | No concerns | Some concerns | Some concerns | Major concerns | Very low |

| Table S8. Confidence in the evidence for each treatment comparison for dynamic balance | | | | | | | | |
| --- | --- | --- | --- | --- | --- | --- | --- | --- |
| Comparison | Number of studies | Within-study bias | Reporting bias | Indirectness | Imprecision | Heterogeneity | Incoherence | Confidence rating |
| Mixed evidence | | | | | | | | |
| AC:BTSNVR | 1 | Some concerns | Low risk | No concerns | Some concerns | No concerns | Major concerns | Very low |
| AC:Kinect | 4 | Some concerns | Low risk | No concerns | No concerns | No concerns | No concerns | Moderate |
| AC:UC | 3 | Some concerns | Low risk | No concerns | No concerns | Some concerns | No concerns | Low |
| AC:Wii | 5 | Some concerns | Low risk | No concerns | No concerns | No concerns | No concerns | Moderate |
| BioRescue:UC | 1 | Some concerns | Low risk | No concerns | No concerns | Major concerns | Major concerns | Very low |
| IVR:UC | 1 | Some concerns | Low risk | No concerns | No concerns | Major concerns | Major concerns | Very low |
| Kinect:UC | 4 | Some concerns | Low risk | No concerns | No concerns | Some concerns | No concerns | Low |
| UC:Wii | 2 | Some concerns | Low risk | No concerns | No concerns | Some concerns | No concerns | Low |
| Indirect evidence | | | | | | | | |
| AC:BioRescue | 0 | Some concerns | Low risk | No concerns | Some concerns | No concerns | Major concerns | Very low |
| AC:IVR | 0 | Some concerns | Low risk | No concerns | Some concerns | No concerns | Major concerns | Very low |
| BTSNVR:BioRescue | 0 | Some concerns | Low risk | No concerns | Some concerns | No concerns | Major concerns | Very low |
| BTSNVR:IVR | 0 | Some concerns | Low risk | No concerns | Some concerns | No concerns | Major concerns | Very low |
| BTSNVR:Kinect | 0 | Some concerns | Low risk | No concerns | Some concerns | No concerns | Major concerns | Very low |
| BTSNVR:UC | 0 | Some concerns | Low risk | No concerns | No concerns | Some concerns | Major concerns | Very low |
| BTSNVR:Wii | 0 | Some concerns | Low risk | No concerns | Some concerns | No concerns | Major concerns | Very low |
| BioRescue:IVR | 0 | Some concerns | Low risk | No concerns | Major concerns | No concerns | Major concerns | Very low |
| BioRescue:Kinect | 0 | Some concerns | Low risk | No concerns | Some concerns | No concerns | Major concerns | Very low |
| BioRescue:Wii | 0 | Some concerns | Low risk | No concerns | Some concerns | No concerns | Major concerns | Very low |
| IVR:Kinect | 0 | Some concerns | Low risk | No concerns | Some concerns | No concerns | Major concerns | Very low |
| IVR:Wii | 0 | Some concerns | Low risk | No concerns | Some concerns | No concerns | Major concerns | Very low |
| Kinect:Wii | 0 | Some concerns | Low risk | No concerns | No concerns | No concerns | Major concerns | Very low |

| Table S9. Confidence in the evidence for each treatment comparison for fear of falling | | | | | | | | |
| --- | --- | --- | --- | --- | --- | --- | --- | --- |
| Comparison | Number of studies | Within-study bias | Reporting bias | Indirectness | Imprecision | Heterogeneity | Incoherence | Confidence rating |
| Mixed evidence | | | | | | | | |
| AC:BTSNVR | 1 | Some concerns | Low risk | No concerns | No concerns | No concerns | No concerns | Moderate |
| AC:IVR | 1 | Some concerns | Low risk | No concerns | No concerns | No concerns | No concerns | Moderate |
| AC:Kinect | 2 | Some concerns | Low risk | No concerns | No concerns | No concerns | No concerns | Moderate |
| Kinect:UC | 2 | Some concerns | Low risk | No concerns | No concerns | No concerns | No concerns | Moderate |
| UC:Wii | 2 | Some concerns | Low risk | No concerns | No concerns | No concerns | No concerns | Moderate |
| Indirect evidence | | | | | | | | |
| AC:UC | 0 | Some concerns | Low risk | No concerns | No concerns | No concerns | No concerns | Moderate |
| AC:Wii | 0 | Some concerns | Low risk | No concerns | No concerns | No concerns | No concerns | Moderate |
| BTSNVR:IVR | 0 | Some concerns | Low risk | No concerns | No concerns | No concerns | No concerns | Moderate |
| BTSNVR:Kinect | 0 | Some concerns | Low risk | No concerns | No concerns | No concerns | No concerns | Moderate |
| BTSNVR:UC | 0 | Some concerns | Low risk | No concerns | No concerns | No concerns | No concerns | Moderate |
| BTSNVR:Wii | 0 | Some concerns | Low risk | No concerns | No concerns | No concerns | No concerns | Moderate |
| IVR:Kinect | 0 | Some concerns | Low risk | No concerns | No concerns | No concerns | No concerns | Moderate |
| IVR:UC | 0 | Some concerns | Low risk | No concerns | No concerns | No concerns | No concerns | Moderate |
| IVR:Wii | 0 | Some concerns | Low risk | No concerns | No concerns | No concerns | No concerns | Moderate |
| Kinect:Wii | 0 | Some concerns | Low risk | No concerns | No concerns | No concerns | No concerns | Moderate |

| Table S10. Results of pairwise meta-analyses and potential sources of heterogeneity | | | | |
| --- | --- | --- | --- | --- |
| Outcome / Comparison | No. of studies | Heterogeneity (I²) | Pooled effect  (95% CI) | Potential sources of heterogeneity |
| Static Balance |  |  |  |  |
| Kinect VS Control | 3 | 0 | 3.06（1.84,4.29） |  |
| Wii VS Control | 4 | 40% | 2.46（0.75，4.18） | History of falls and limited walking ability |
| Dynamic Balance |  |  |  |  |
| Kinect VS Control | 6 | 66% | -0.29  （-0.53，-0.06） | Balance impairment |
| Wii VS Control | 5 | 58% | -0.35  （-0.83，0.12） | Balance impairment |
| Fear of falling |  |  |  |  |
| Kinect VS Control | 4 | 61% | -0.55  （-0.97，-0.13） | Balance impairment |
| Wii VS Control | 2 | 0 | -0.19  （-0.35，-0.04） |  |
